# Care in Europe after presenting to the emergency department with a seizure; position paper and insights from the European Audit of Seizure Management in Hospitals

**DOI:** 10.1111/ene.15336

**Published:** 2022-05-07

**Authors:** Claire Taylor, Catrin Tudur‐Smith, Pete Dixon, Christine Linehan, Aleksei Gunko, Jakob Christensen, Mike Pearson, Torbjorn Tomson, Anthony Marson, the ESBACE consortium and EuroNASH collaborators, Eugen Trinka, Helene Visee, Chantal Depondt, Susana Ferrao Santos, Annelies Van Dycke, Wim Van Paesschen, Cara Conway, Geraldine O’Rourke, Cora Flynn, Allan McCarthy, Denise Cunningham, Abel Wakai, Masha Petricevic, Daniel Costello, Johan Zelano, Firal Al‐Jasami, Sara Melin, Mans Berglund, Lisa Bergstrom, Victor Vall, Rob Rouhl, Nicola Dingli, Sylvain Rheims, Karim Tazarourte, Louise Tyvaert, Phillipe Derambure, Marie Girot, Cecile Marchal, Eivind Kolstad, Silje Holt Jahr, Siri Hylleraas Bo, Christian Samsonsen, Reetta Kalvianen, Maria Mazurkiewicz‐Beldzinska, Igor Kaymovskiy, Phillipe Ryvlin, Ilona Wisiewski, Celia Bader, Felix von Podewils, Susanne Knake, Katja Menzler, Martin Hirsch, Jochen Brich, Rainer Surges, Karmele Olaciregui Dague, Tim Wehner, Adam Strzelczyk, Lara Kay, Lea Miklic, Vasilios Kimikidis, Martha Spilioti, Theodora Afrantou, Aikaterina Terzoudi, Dimitrios Kazis, Sofia Markoula, Katerina Markou, Savvas Papacostas, Yiolanda Panayiota Christou

**Affiliations:** ^1^ Institute of Population Health, University of Liverpool Liverpool UK; ^2^ UCD Centre for Disability Studies University College Dublin Dublin Ireland; ^3^ Department of Neurology Aarhus University Hospital Aarhus Denmark; ^4^ 1006 Department of Clinical Medicine Aarhus University Aarhus Denmark; ^5^ 27106 Department of Clinical Neuroscience Karolinska Institutet Stockholm Sweden; ^6^ Department of Pharmacology and Therapeutics, University of Liverpool Liverpool UK

**Keywords:** audit, epilepsy, neurological disorders, quality improvement, service evaluation

## Abstract

**Background and purpose:**

This position paper makes recommendations following an audit of care provided to people presenting with a seizure to emergency departments (EDs) in Europe.

**Methods:**

Participating countries were asked to include five hospitals agreeing to identify 50 consecutive seizure patients presenting to their ED between 1 August 2016 and 31 August 2017. Anonymous data were collected to a web database. Where quoted, percentages are mean site values and ranges are the 10th–90th centile.

**Results:**

Data were collected on 2204 ED visits (47 sites, up to six per country, across 15 countries): 1270 (58%) known epilepsy, 299 (14%) previous blackouts but no epilepsy diagnosis, 634 (29%) with a first seizure. Wide variability was identified for most variables. Of those with known epilepsy, 41.2% (range 26.2%–59.6%) attended the ED in the previous 12 months, but only 64.7% (range 37.2%–79.8%) had seen an epilepsy specialist in the previous 12 months. 67.7% (range 34.0%–100%) were admitted, 53.1% to a neurology ward (range 0.0%–88.9%). Only 37.5% first seizure patients (range 0.0%–71.4%) were given advice about driving.

**Conclusions and recommendations:**

It is recommended that in Europe guidance is agreed on the management and onward referral of those presenting to the ED with a seizure; a referral process is created that can be easily implemented; it is ensured that the seizure services receive referrals and see the patients within a short time period; and a simple system is developed and implemented to allow continuous monitoring of key indices of epilepsy care.

## Graphical abstract

The European Audit of Seizure Management in Hospitals (EuroNASH) shows that there is considerable and unacceptable variability in the quality of care provided to people presenting to emergency departments with a seizure in Europe. In this position paper it is recommended that in Europe (1) guidance is created and agreed for specialist referral; (2) a process for referral is created that is easy to do and can be incorporated into the workings of local hospitals with minimal effort; (3) it is ensured that the epilepsy/seizure service receives these referrals and sees the patients within a short time period; (4) a simple system is developed and implemented to allow continuous monitoring of key indices of epilepsy care.

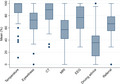

## INTRODUCTION

Neurological diseases are common across Europe but often do not get political attention so the needs of those with neurological conditions and the investment in services, training and education are often overlooked [1]. Whilst there is a growing campaign highlighting the burden of neurological diseases in Europe [2], less attention has been paid to the coordination of, access to and quality of clinical services aiming to reduce that burden.

Most epilepsy research has focused on the development of treatments and interventions, but few clinical trials have assessed service level interventions [3], and there have been few assessments of the process of care and related outcomes, and costs [4, 5]. When doing so, it is important to consider the wider population, as it is possible that those outside specialist care have worse outcomes [6], which could be improved if ambulatory care was more accessible.

In this paper, the focus is on the coordination of care for one emergency neurological presentation, seizure, across Europe, highlighting inadequacies in care based on the results of audit. Recommendations are made for improving care in the context of a quality improvement framework, learning from programmes of quality improvement in other clinical disciplines.

Seizures are a common presentation: one in 10 people will have a seizure in their life and the prevalence of epilepsy is about 0.6% [7]. Good care can reduce adverse outcomes, although it has been estimated that around 50% of people with epilepsy are currently rendered seizure free, whilst evidence suggests that this should be 70%–80% [8]. This treatment gap is not only a problem of access to anti‐seizure medications but also reflects a lack of access to expertise and poor coordination of care [9].

Seizures account for about 1% of medical (i.e., not surgical or obstetric etc.) admissions [10, 11], with a similar number attending the ED but without admission. The UK National Audit of Seizure Management in Hospitals (NASH) has had two rounds of audit covering almost 9000 individuals from more than 150 hospitals. It found that performance of even relatively simple aspects of care varied widely; some sites performed consistently well, demonstrating that providing care to standards set in national guidelines is possible, but in others tasks such as obtaining a witness history are often not done and routine assessments are inexplicably missing [12].

Our overarching aim is to provide data that can inform programmes of quality improvement in epilepsy care, leveraging from experience in other clinical disciplines. For example, collecting reliable up‐to‐date data on ‘time to needle’ in the UK led to the proportion of acute myocardial infarctions receiving thrombolysis within 30 min rising from less than 35% to nearly 80% with substantial benefit in survival outcomes [13]. This improvement was enabled by the Central Cardiac Audit Database led from the Royal College of Physicians, established in 1998 to measure the ‘time to needle’. Cardiologists, ambulance teams and patient organizations collaborated to establish a minimum dataset needed to address the question; each hospital entered their own data and had available a comparative report on a daily basis showing performance over time and in comparison to neighbouring sites. In just over 2 years all UK hospitals were participating. Success factors included (i) shared ‘buy in’ from professionals and patient groups, (ii) the introduction of thrombolysis nurses and (iii) instant feedback in the public domain. That project has continued and successfully expanded to cover other aspects of cardiology [13]. There were similar achievements in stroke care supported by national audit, leading to the creation of stroke units across the country, with benefits in outcomes [14].

In 2011, these achievements led some of the current investigators to wonder if it would be possible to apply these approaches to epilepsy and the UK NASH was born. A seizure presenting to hospital was the starting point from which to examine care immediately before, during and following the event. Then in 2011 and 2013, NASH had two rounds of audit covering almost 9000 individuals from more than 150 hospitals. Performance of even relatively simple aspects of care varied widely. Some sites performed consistently well, demonstrating that providing care to standards set in national guidelines is possible, but in others even simple tasks such as obtaining a witness history are often not done and routine assessments are inexplicably missing [12].

A third round of NASH in 2019 showed that across 137 hospitals there had been very little change [15]. This was hugely disappointing and raises many questions about what could be done to stimulate better care. Particular issues are that many patients are not under specialist review and do not get referred for a review after a hospital presentation—and so the opportunity to revise therapy and prevent further episodes is not being offered, and the huge variability across sites observed in 2011 and 2013 continues.

It is important to assess whether these problems in the coordination of epilepsy care are peculiar to the UK or are also prevalent in other European countries. The European Union funded European Study of Burden and Care in Epilepsy (ESBACE) provided an opportunity to address this question, one of the objectives of which was to provide information on the coordination of services via the European Audit of Seizure Management in Hospitals (EuroNASH). The design of EuroNASH was modelled on the UK study with the intention first of demonstrating that multi‐country audit is feasible and second of examining how variable care is across Europe. Finally, it was hoped that the audit might be a means of identifying, from the best, some opportunities to improve care more widely.

## METHODS

### Countries

All countries affiliated to the International League Against Epilepsy (ILAE) Europe were invited to take part in EuroNASH via communication from the ILAE Europe secretariat and at a meeting of the representatives of the national ILAE chapters at the 2016 European Epilepsy Congress where one author (AGM) gave a presentation to encourage participation. For each participating country a national coordinator was identified who was asked to identify five hospitals in their country to participate.

### Recruitment of cases

Each hospital was asked to provide information on 50 consecutive cases presenting to the ED with a seizure between 1 August 2016 and 31 August 2017. Data entry opened in March 2017 and closed in February 2018. Data collection was retrospective to ensure 3‐month follow‐up information could be available. If a person attended the same ED more than once, each attendance was treated as a separate event. A proforma was developed to collect data, based on that used in NASH, which was adapted to a wider European setting by the steering committee and ESBACE consortium. The proforma had 10 sections that covered acute, prior and onward care. A bespoke web‐based data entry system was developed to allow sites to enter anonymous data on a secure server in Liverpool.

Questions enabled the identification of three subgroups of patients: those with a previous epilepsy diagnosis (group 1), those with previous blackouts or seizures but no definite epilepsy diagnosis (group 2) and those with a likely first seizure (group 3). Analyses compared performance across sites and data were summarized as median and the 10th–90th percentile range. For inter‐country comparisons, eight countries with multiple sites were compared. The seven countries with only one site were grouped as ‘other countries’.

As per the NASH audits [16], the widest possible interpretation of care delivery was allowed for so, for example, any contact with one of the listed specialists (neurologist, epilepsy specialist nurse, paediatrician etc.) would be classed as contact with an epilepsy specialist.

## RESULTS

### Hospital participation

Fifteen of the 39 European countries invited to do so by ILAE Europe participated. Eight countries recruited multiple sites (three to six sites, total 40 sites), whilst seven had one site each. Reasons (when given) for not taking part included a lack of personnel to do the audit (five countries), unavailability of funding (one country) and concerns over information governance (four countries). Of the participating sites, the great majority were urban (94%) and academic or teaching hospitals (90%). Data were collected by doctors (80.6%), nurses (8.1%) and by others (11.3%) including other health professionals, clinical audit staff and medical students.

### Demographics of participants included in the audit

Data were collected on 2204 ED attendances, median age 49 years (interquartile range 30–66; complete range 17–98 years), 56% were male (Table 1). There were 1270 (58%) with an existing epilepsy diagnosis (group 1), 299 (14%) with previous blackouts or seizures but no definite epilepsy diagnosis (group 2) and 634 (29%) with a likely first seizure (group 3). One person could not be classified into one of the three patient types. Across the sites, there are significant differences in age (*p* < 0.01), gender (*p* = 0.06) and the proportion with established epilepsy (*p* < 0.01).

**TABLE 1:** Demographics and patient groups in the participating countries and sites

Country	Sites (*n*)	Patients (*n*)	Age (median, interquartile range)	Gender (% male)	Group 1: existing epilepsy diagnosis (*n*, %)	Group 2: previous blackouts or seizures, no definite epilepsy diagnosis (*n*, %)	Group 3: likely first seizure (*n*, %)
Countries with multiple sites							
Ireland	6	300	37.5 (27–52.5)	53.3	192 (64.0)	36 (12.0)	72 (24.0)
Sweden	6	300	47 (26–68)	48.3	187 (62.3)	52 (17.3)	61 (20.3)
Germany	6	268	56 (34.5–76)	52.6	121 (45.1)	44 (16.4)	103 (38.4)
Greece	5	254	45 (30–61)	61.8	142 (55.9)	39 (15.4)	73 (28.7)
Belgium	5	252	52 (33–66)	61.5	150 (59.5)	21 (8.3)	81 (32.1)
Austria	5	127	55 (38–70)	63.8	101 (79.5)	4 (3.1)	22 (17.3)
France	4	199	49 (33–63)	55.8	100 (50.5)	35 (17.7)	63 (31.8)
Norway	3	153	56 (34–71)	59.5	83 (54.2)	23 (15.0)	47 (30.7)
Total	40	1853	49 (30–66)	56.2	1076 (58.1)	254 (13.7)	522 (28.2)
Countries with single sites							
Croatia	1	50	49.5 (35–75)	48.0	28 (56.0)	10 (20.0)	12 (24.0)
Cyprus	1	51	34 (27–52)	54.9	24 (47.1)	8 (15.7)	19 (37.3)
Finland	1	50	64.5 (39–72)	60.0	19 (38.0)	10 (20.0)	21 (42.0)
Malta	1	50	47 (32–60)	60.0	27 (54.0)	2 (4.0)	21 (42.0)
Netherlands	1	50	53 (34–66)	48.0	20 (40.0)	12 (24.0)	18 (36.0)
Poland	1	50	41.5 (25–57)	52.0	36 (72.0)	1 (2.0)	13 (26.0)
Switzerland	1	50	54 (33–67)	64.0	40 (80.0)	2 (4.0)	8 (16.0)
Total	7	351	49 (30–66)	55.3	194 (55.3)	45 (12.8)	112 (31.9)

### Emergency department and neurology clinic attendance in the previous year and current antiseizure medication (ASM) therapy

Out of the 2204 ED attendees, 602 (27.3%) had attended the ED for epilepsy in the previous 12 months (range 14.0%–40.0%), 1030 (46.7%) had been seen in a neurology or seizure clinic in the previous 12 months (range 25.9%–64.0%) and 1194 (54.2%) were on ASMs prior to this event (range 35.5%–72.0%). In each instance the proportions were higher in group 1 patients (those with a known epilepsy diagnosis) than in groups 2 and 3 (Table 2).

**TABLE 2:** Care prior to the current seizure episode across all 47 sites

	Group 1 (*n* = 1270)		Group 2 (*n* = 299)		Group 3 (*n* = 634)	
	Mean %	Range	Mean %	Range	Mean %	Range
Reviewed by a specialist in previous year	64.7	37.2–79.8	34.1	0.0–66.7	16.7	0.0–33.3
Reviewed by an adult or paediatric neurologist in previous year	61.4	29.8–78.4	30.1	0.0–65.3	11.5	0.0–26.6
Attended ED or admitted in previous year with seizure	41.2	26.2–59.6	24.1	0.0–50.0	1.1	0.0–1.9
Taking ASMs	88.3	72.4–97.0	11.0	0.0–48.0	6.2	0.0–12.4
Taking ASM as monotherapy	48.7	36.8–63.1	10.7	0.0–48.0	5.4	0.0–11.7

Note

In each case the data show the mean site value and the 10–90 percentile range across the 47 sites.

Abbreviations: ASM, antiseizure medication; EM, emergency department.

The most commonly prescribed ASM in treatment regimens across European sites for those with a previous epilepsy diagnosis was levetiracetam (38.8%; range 22.2%–55.9%), followed by valproate (24.8%; range 8.3%–41.4%), lamotrigine (22.0%; range 3.7%–37.5%) and carbamazepine (13.2%; range 2.5%–25.0%). In all sites there were patients with established epilepsy (group 1) taking the older drugs phenobarbitone (2.8%), phenytoin (5.6%) or valproate (24.8%) as monotherapy.

### Acute seizure management prior to and on arrival in the ED

Rescue medication was administered prior to ED attendance (by carer, ambulance staff or general practitioner) across European sites to 269/1270 (21.2%) group 1 patients (range 0.0%–42.9%), 22/299 (7.4%) group 2 patients (range 0.0%–20.0%) and 88/634 (13.9%) group 3 patients (range 0.0%–31.6%). Of those given rescue medication, it included diazepam (rectal or intravenous) for just over half (50.1% [0%–100%]) and buccal or intravenous, intramuscular midazolam for 38.0% (0%–90.9%). It was also identified that, whilst guidelines such as the National Institute for Health and Care Excellence epilepsy guidelines recommend buccal midazolam [16], it is not available in some European countries.

In 254 (11.5%, range 2.4%–21.9%) patients, seizures were ongoing at arrival at the ED for whom the most commonly used therapies given in the ED were intravenous diazepam 40.6% (range 0%–77.8%), intravenous lorazepam 22.1% (range 0%–85.7%) and intravenous valproate 13.4% (range 0%–46.2%).

### Management in the hospital and assessments in the ED

**FIGURE 1:**
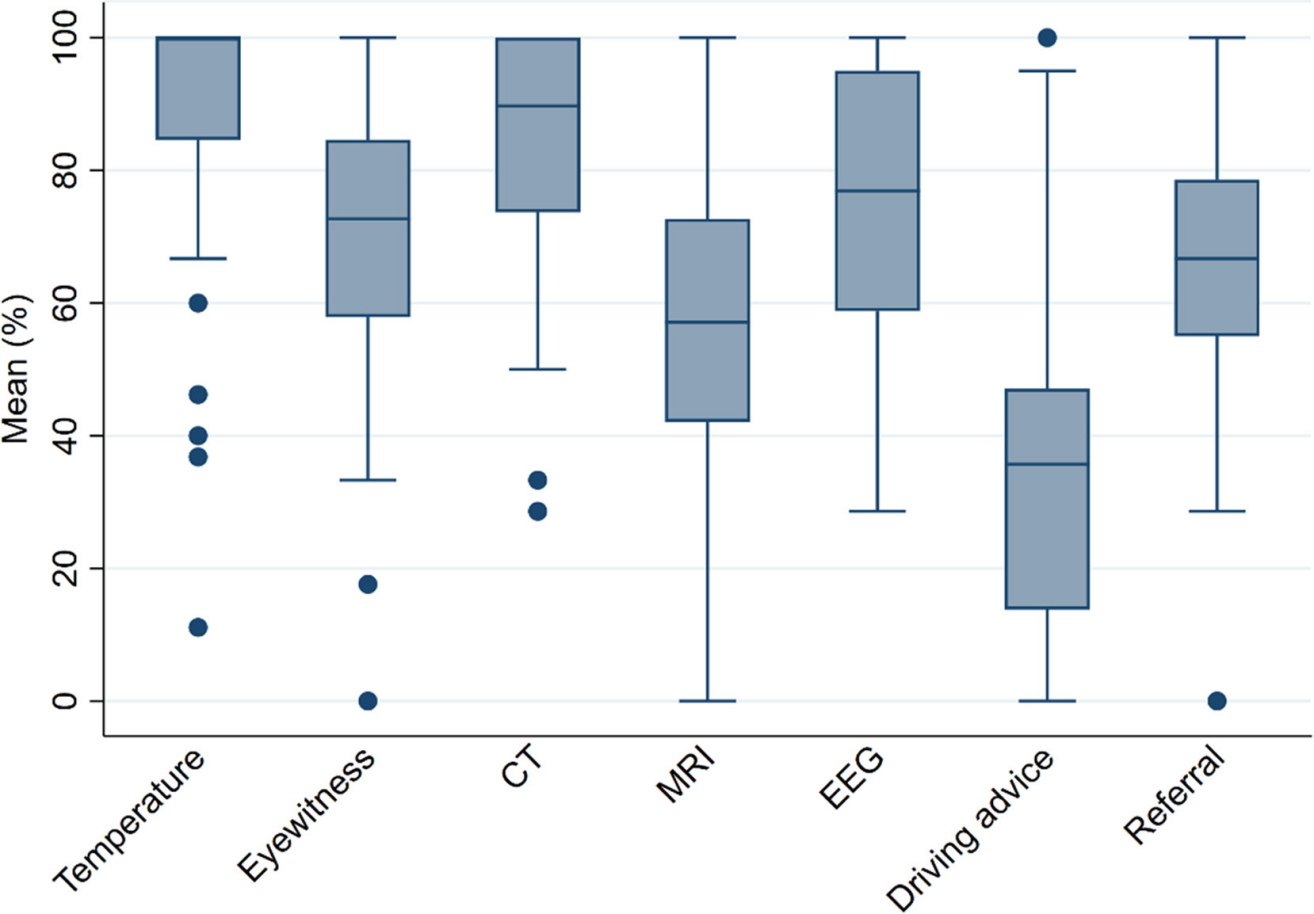
Variation between sites in undertaking investigations for group 3 (likely first seizure patients). Box and whisker plot showing median of site means, 25th and 75th centiles and range [Colour figure can be viewed at wileyonlinelibrary.com]

Details of care delivered on arrival at the ED, the investigations requested and the ongoing treatment plan are summarized in Table 3 for each of the three patient groups and illustrated in Figure 1 for those in group 3. There was variation in the recording of basic assessments such as temperature (e.g., group 1 range of site means 45.0%–100%), as well as variation in performing elements of the neurological examination including testing the plantar reflexes (e.g., group 1 range of site means 27.6%–100.0%) and fundoscopy (e.g., group 1 range of site means 0%–60.0%). Even for likely first seizure patients (group 3), where serious intracranial pathology has to be excluded and a neurological examination undertaken, recording the plantar reflex ranged from 30.0% to 100.0% and fundoscopy from 0% to 77.8%. There was also variation in the recording of an attempt to gain an eyewitness account where even for group 3 (first seizure patients) it was 40.0%–91.7%. Simple investigations (electrocardiography, ECG) were not recorded for more than half of group 3 patients in some sites.

**TABLE 3:** Assessment, investigation and onward management after presenting to the ED with a seizure for the three subtypes of presentation

	Group 1 (*n* = 1270)		Group 2 (*n* = 299)		Group 3 (*n* = 634)		All patients (*n* = 2204)	
	Mean (%)	Range	Mean (%)	Range	Mean (%)	Range	Mean (%)	Range
Temperature	85.6	45.0–100.0	90.3	33.3–100.0	89.6	60.0–100.0	87.4	54.0–100.0
Fundoscopy	14.3	0.0–60.0	27.4	0.0–80.0	22.1	0.0–77.8	18.3	0.0–70.0
Plantar reflex	66.1	27.6–100.0	75.9	50.0–100.0	77.4	30.0–100.0	70.7	30.6–100.0
Witness history	57.8	32.4–83.9	69.9	33.3–100.0	72.1	40.0–91.7	63.5	41.4–84.0
Blood glucose	89.2	62.5–100.0	93.3	80.0–100.0	95.1	85.7–100.0	91.5	68.0–100.0
ECG	63.8	21.4–100.0	81.3	33.3–100.0	82.2	40.0–100.0	71.4	32.0–100.0
EEG	40.6	9.7–95.0	70.6	33.3–100.0	76.7	45.5–100.0	55.0	26.0–93.5
CT	44.1	20.0–76.5	65.6	22.2–100.0	85.8	60.0–100.0	59.0	33.3–80.0
MRI	17.5	3.5–35.0	52.8	0.0–90.0	56.8	22.2–85.7	33.6	16.0–60.0
Discharged directly	38.0	0.0–72.5	32.4	0.0–83.3	18.5	0.0–58.3.0	31.6	0.0–66.0
Driving advice	21.6	0.0–59.3	47.7	0.0–100.0	37.5	0.0–71.4	29.6	0.0–56.6
Admitted	61.2	27.5–100.0	66.2	11.1–100.0	81.4	41.7–100.0	67.7	34.0–100.0
Advice re future seizures	30.8	0.0–89.2	22.8	0.0–85.7	24.8	0.0–81.3	28.0	2.0–88.0
Referral to neurology	74.2	55.0–96.3	81.9	50.0–100.0	67.5	35.7–89.5	73.3	54.2–90.0

Note

In each case the data show the mean site value and the 10–90 percentile range across the 47 sites.

Abbreviations: CT, computed tomography; ECG, electrocardiography; EEG, electroencephalography; MRI, magnetic resonance imaging.

More complex investigations (electroencephalography [EEG], computed tomography [CT] or magnetic resonance imaging [MRI]) were more likely to be ordered for group 3 patients, for those admitted to hospital and for older patients. For example, 44.1% of those with established epilepsy (group 1) had CT scans requested, including 55.9% of those admitted versus 25.9% of those discharged directly from the ED, and CT scans were requested in 60.9% of those aged over 60 versus 37.7% aged under 60 years.

There was substantial variation in the percentage admitted (e.g., group 1 range 27.5%–100% and group 3 range 41.7%–100%) and in the ward type they were admitted to (e.g., in group 1, 0%–93.5% were to a neurology ward). The median length of stay was 3 days (range 1–11 days) for both first seizure (group 3) patients and those with an existing epilepsy diagnosis (group 1).

Data collection on advice given to patients about driving or the management of future seizures allowed for the widest definition; for example, the advice on driving standard was based on establishing either that the person was a non‐driver or that advice was given to drivers. Even with this broad definition there is still wide variation across sites (e.g., group 3 mean 37.5%, range 0%–71.4%).

Referral for specialist neurology input included both inpatient assessment and referral to outpatient services. For group 3 (first seizure patients) this varied between 35.7% and 89.5% across sites. Referral rates were higher for group 1 (55.0%–96.3%) and group 2 (50.0%–100.0%) patients. Patients were more likely to be referred if younger (79.0% aged under 60 vs. 62.3% if older) and if they were recorded as having had an alcoholic binge (83.5% vs. 72.6%).

Variability amongst sites is further illustrated in Figure 1, which shows results for recording temperature, contacting an eyewitness, CT and MRI imaging, driving advice and making a specialist referral.

### National versus site variation

**FIGURE 2:**
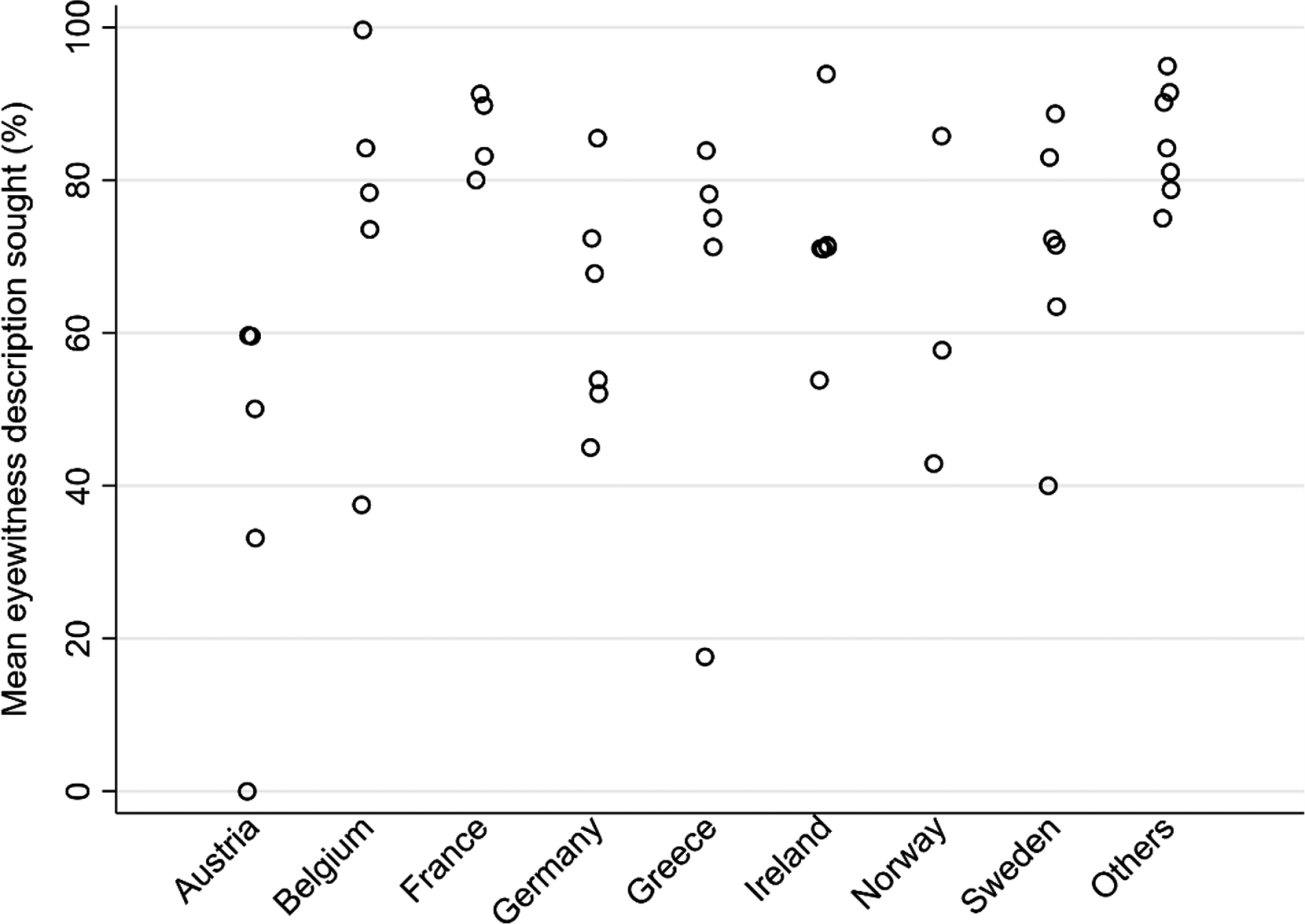
Variation in the seeking of a witness description in first seizure presentations (group 3) across countries—the seven countries with one site each appear as ‘other’

**FIGURE 3:**
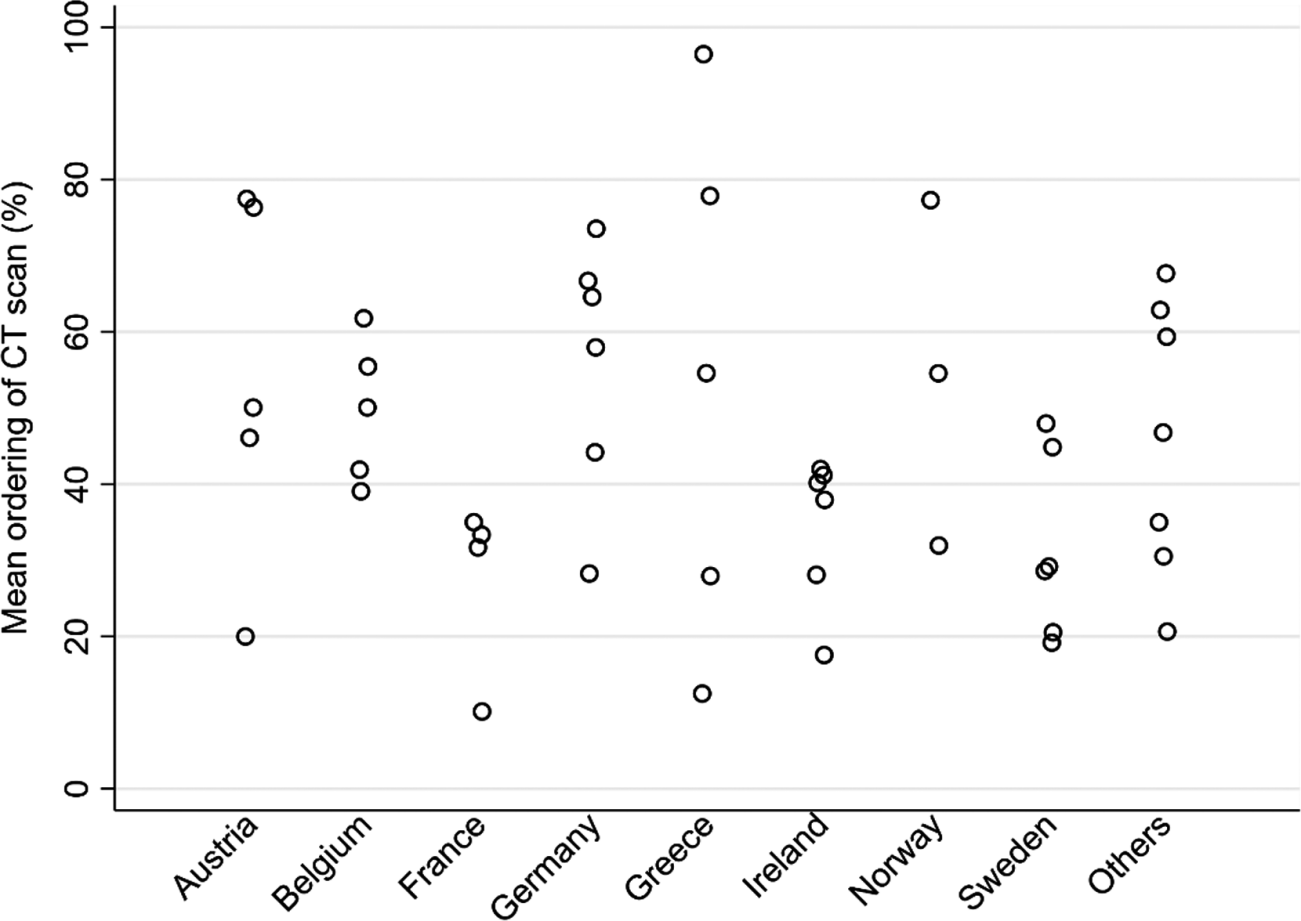
Variation by country and site as to whether a CT scan was ordered after a seizure presentation in group 1 patients

**FIGURE 4:**
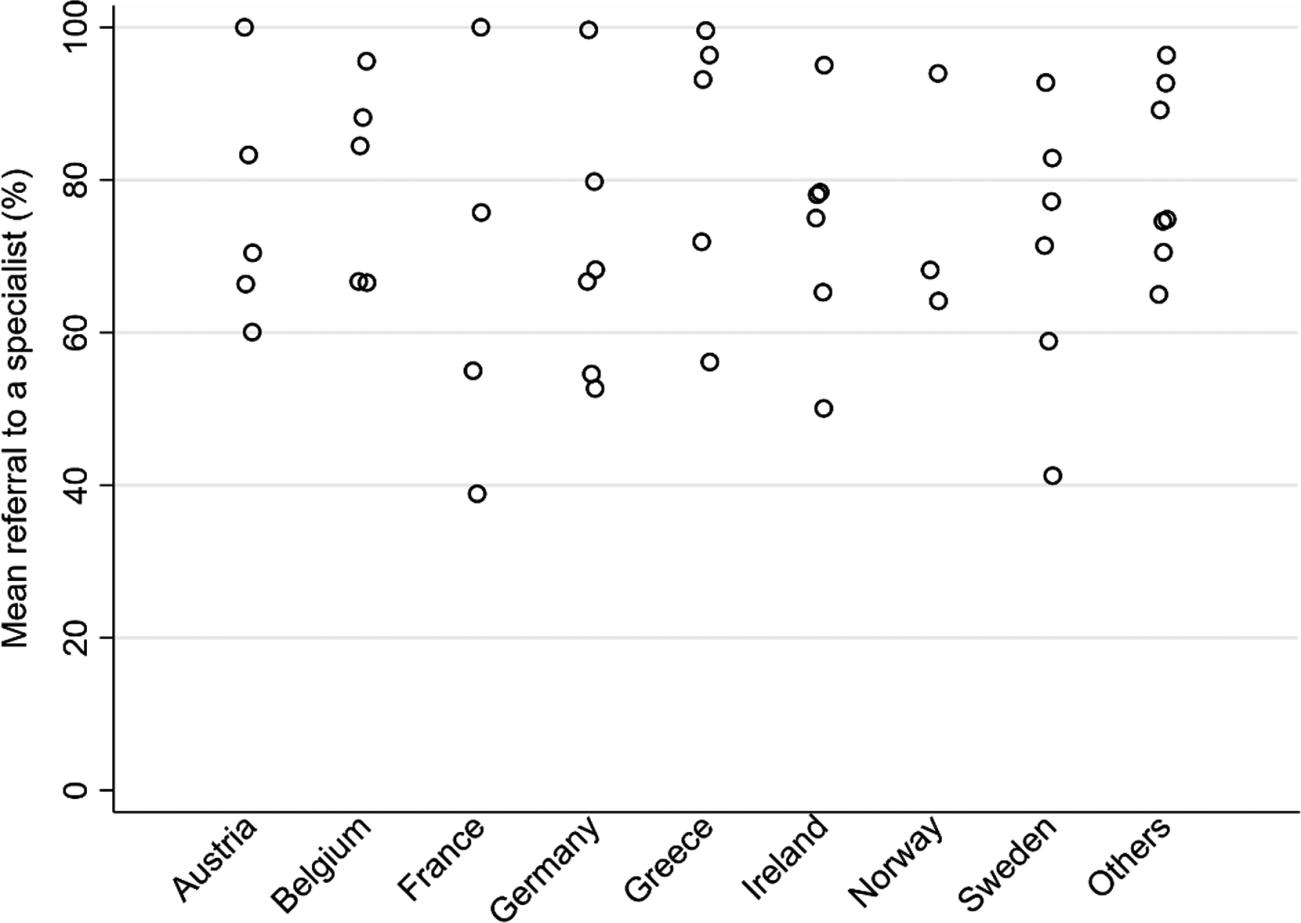
Variation by country and site of proportions referred for a specialist opinion (group 1)

Variation is apparent not just amongst sites but also amongst countries, which was present for all three patient groups for most of the recorded variables, and is illustrated for three examples in Figures 2,3 and 4. Due to the small number of sites per country, no attempt was made to calculate the relative inter‐country and intra‐country variability.

For the variables illustrated in Figures 2, 3, 4, it is clear that there is wide variability in performance within individual countries and between sites.

## DISCUSSION

### Does the UK experience apply to the rest of Europe?

EuroNASH used the same method as the UK NASH project [12] and shows similar findings across Europe for those presenting to the ED with a seizure. The range of care quality is wide and many basic aspects of care are not being done. It was also clear that an international audit of adult epilepsy care is feasible.

The strengths of the study include that the data collection tool was based on an established online tool and that over 85% of data were collected by clinical professionals, suggesting positive local clinical ‘buy in’ to the project. Although launched at a point when there were widespread concerns about General Data Protection Regulations [17] (and several sites opted out because they were unsure) it has been possible to perform this study collecting fully anonymized data.

The observed variability between sites is very wide across all aspects of care, similar in magnitude to that reported by UK NASH [12] and so large as to be well beyond any chance effect. Items such as recording of temperature on arrival should be routine in any ED, and an eyewitness history is vital when making a diagnosis of seizure and it is therefore worrying that there was no attempt to gain one for a quarter of first seizure cases. It is hard to justify why simple tests such as the blood sugar or ECG are missing in significant numbers of cases—except to note that such errors of omission have been common to many national audits [18].

There was also considerable variation in the use of inpatient care, one of the costliest resources, the percentage of patients admitted ranging from 34% to 100% across sites, mean stay 5.1 days (range 1–11). It is unlikely that clinical need explains this variability and clear guidance is required to determine when admission would be an efficient use of healthcare resources.

Whilst, as expected, CT brain imaging was more commonly undertaken for first seizure presentations, there was considerable variability amongst sites (range 60%–100%). Variability was even greater for those with known epilepsy (20%–76.5%), the majority of whom will have had previous brain imaging and for whom the likely utility of an acute CT brain scan is low. These results mirror the UK NASH audit, where the main factor associated with CT head scanning was the hospital the patient attended rather than clinical characteristics of the patient [19]. Similarly, EEG was undertaken in 40.6% of those with known epilepsy (range 9.7%–95.0%), but it is very difficult to see how most EEG in those with a known epilepsy diagnosis would have had any impact on patient management. These findings highlight a clear need for guidance on the use of brain imaging and EEG in the acute setting.

There were also inadequacies in the recording of advice given to patients and carers about the management of future seizures should they occur, and on advice given about driving, for which there are clear European standards. The variability in the rate of onward referral to neurology services for onward care is also of concern, as it is from epilepsy and neurology services that patients should get the best care and advice in order to reduce the risk of future seizures.

### What can epilepsy learn from other specialities?

The evidence that guidelines alone will change practice is limited [20]. Examples from cardiac and stroke care began with setting a limited number of targets that were agreed across the relevant professionals and patient groups and some solutions to help meet those targets. The data collection and analysis identified where change was most needed and whether it was happening. Comparative data in the public domain that was professionally owned proved a strong incentive to participate. The changes in care for cardiology and stroke care were not a simple change of clinician behaviour, but rather the introduction of new systems of care, that is, thrombolysis nurses to implement the rapid therapy needed on arrival and stroke units bringing together the team that delivers the care. Thus, the environment in which the clinicians were operating changed and that affected the way the clinicians functioned, which has also been shown elsewhere [21]. All UK hospitals were taking part within 2 years and the ‘time to needle in 30 min’ target was reached in nearly 80% of cases with a significant reduction in 30‐day mortality [13]. Similar trends have been observed in other European countries [22, 23].

The Central Cardiac Audit Database was a world leader and continues to operate, but it depends on each local unit sending in all their data on a regular basis. The stroke audit’s approach of a 2‐yearly repeat data collection was simpler to run but associated with a much slower pace of change. Stroke was an unfashionable topic at the time but it was a common cause of medical admission and there was a strong lobby of interested physicians and patient charities. Over the first 8 years, however, the National Health Service went from 25% of hospitals having a stroke unit to all and each led by a stroke physician. The cycle of repeat audit studies showing beneficial outcomes was useful as justification to those managing the service to support change [24].

This cycle was the process adopted by the UK NASH project although thus far there have been very modest changes. So, whilst the audits in cardiology and stroke were a trigger for change and a means of monitoring change, there is a need to define what is needed for an epilepsy and seizure care service.

### Can this approach be applied to epilepsy and seizure services?

There are several reasons why clinicians and those managing services may be less likely to prioritize seizures and epilepsy compared to other chronic disorders. Seizures are less common than heart attacks or strokes presenting to hospital and therefore less costly. Seizures are a less dramatic presentation in most cases as the seizure has ended by the time the patient arrives in the ED. There are no monitoring tests or specific tasks to justify regular review outside of specialist care compared with, for example, diabetes. The neurology specialist services are not always available as part of emergency or acute care and may reside at another site or specialist hospital. This creates a sense of ‘distance’ between those in the emergency department front line, for example, and the speciality. It also means that there is no champion on site to ensure that the needs of epilepsy and seizures are considered in the planning or service structures.

However, there is clearly a need to improve the coordination and delivery of epilepsy and seizure care across and within European countries. Clinicians and patient groups will therefore need to make a strong case for attention to and investment in service provision.

Large national audits have been a vehicle to improved care in the UK, and one of the important learnings is that change takes time and requires a local champion who can get structures and systems in place [25]. One challenge is the disparity between where the patients are and where the expertise is sited. There are benefits from concentrating expertise and diagnostic facilities in fewer sites, but the downside is that expecting people with epilepsy to travel is a barrier. Also the knowledge sharing between the expert centres and healthcare professionals in secondary care and in the community is limited.

Specialist services can provide a firm diagnosis, access to investigations and a management plan that needs to include the use of rescue therapy, as well as what is expected of ambulances and EDs for that individual. Given the EuroNASH findings, it is suggested that first and foremost there must be a reliable referral service to specialist advice. This should apply to those with a first suspected seizure as well as those with known epilepsy. Guidance is also needed on the management and use of investigations for those presenting to the ED with seizures.

A five‐step approach is suggested.

- Create and agree on guidance on the management and use of investigations for those presenting to EDs due to a seizure. This is a multidisciplinary issue that must include collaboration amongst relevant clinical disciplines, managers and patients or representatives. This process should also identify metrics and quality standards against which performance can be assessed [26].
- Create and agree on a guidance document for all hospitals to make specialist referral the norm, using the same multidisciplinary approach as above.
- Create a process by which referral can be achieved that is easy to do and can be incorporated into the workings of local hospitals with minimal effort. Patient issues such as transport must also be considered. Better use of information technology could be a means to achieve this.
- Ensure that the epilepsy clinic service is able to receive these referrals and see the patients within a short time period. In many countries this may have staffing implications. Patients who have had an acute and worrying episode should not have long waits to be seen.
- Develop and implement a simple system to allow continuous monitoring of key indices of epilepsy care, building on our experience with EuroNASH.

Whilst this is easy to state, however, it is much harder to achieve. Change will require leadership, multidisciplinary collaboration, education, systems change, new processes and new ways of working. However, the challenge must be faced if the difference that is so sorely needed is to be made.

## CONFLICT OF INTEREST

Claire Taylor, Pete Dixon, Mike Pearson, Catrin Tudur Smith, Christine Linehan and Alex Gunko have no conflicts of interest to declare. Professor Marson leads the UK Audit of Seizure Management in Hospitals, which is supported by a grant from UCB Pharma to the University of Liverpool. Dr Christensen has received honoraria for serving on the Scientific Advisory Board of Union Chimique Belge (UCB) Nordic and Eisai AB. Dr Christensen has also received honoraria for giving lectures for UCB Nordic and Eisai and received travel funds from UCB Nordic. Dr Tomson reports speakers’ honoraria to his institution from Eisai, Sanofi, Sun Pharmaceutical Industries Ltd and UCB, and research support from Bial, Eisai, GlaxoSmithKline, Stockholm County Council, Teva, GW Pharma, Arvelle and UCB.

## AUTHOR CONTRIBUTIONS

Claire Taylor: Data curation (lead); formal analysis (lead); project administration (lead); writing—review and editing (equal). Catrin Tudur Smith: Data curation (supporting); formal analysis (supporting); methodology (equal); writing—review and editing (equal). Peter Dixon: Data curation (supporting); formal analysis (supporting); funding acquisition (supporting); project administration (supporting); writing—original draft (supporting); writing—review and editing (supporting). Christine Linehan: Funding acquisition (equal); methodology (supporting); writing—review and editing (supporting). Aleksei Gunko: Data curation (supporting); writing—review and editing (supporting). Jakob Christensen: Funding acquisition (equal); methodology (supporting); writing—review and editing (supporting). Michael Pearson: Conceptualization (equal); formal analysis (supporting); methodology (supporting); writing—original draft (equal); writing—review and editing (equal). Torbjörn Tomson: Funding acquisition (equal); methodology (supporting); writing—review and editing (supporting). Anthony Guy Marson: Conceptualization (equal); formal analysis (supporting); funding acquisition (equal); methodology (equal); writing—original draft (equal); writing—review and editing (equal).

## Data Availability

The data that support the findings of this study are available from the corresponding author upon reasonable request.

## EuroNASH Collaborators

Eugen Trinka, Helene Visee, Chantal Depondt, Susana Ferrao Santos, Annelies Van Dycke, Wim Van Paesschen, Aleksei Gunko, Cara Conway/Geraldine O’Rourke, Cora Flynn, Allan McCarthy/Denise Cunningham, Abel Wakai/Masha Petricevic, Daniel Costello, Johan Zelano, Firal Al‐Jasami, Sara Melin, Mans Berglund, Lisa Bergstrom, Victor Vall, Rob Rouhl, Nicola Dingli, Sylvain Rheims/Karim Tazarourte, Louise Tyvaert, Phillipe Derambure/Marie Girot, Cecile Marchal, Eivind Kolstad, Silje Holt Jahr/Siri Hylleraas Bo, Christian Samsonsen, Reetta Kalvianen, Maria Mazurkiewicz‐Beldzinska, Igor Kaymovskiy, Phillipe Ryvlin/Ilona Wisiewski/Celia Bader, Felix von Podewils, Susanne Knake/Katja Menzler, Martin Hirsch/Jochen Brich, Rainer Surges/Karmele Olaciregui Dague, Tim Wehner, Adam Strzelczyk/Lara Kay, Lea Miklic, Vasilios Kimiskidis/Martha Spilioti/Theodora Afrantou, Aikaterina Terzoudi, Dimitrios Kazis, Sofia Markoula, Katerina Markou, Savvas Papacostas/Yiolanda Panayiota Christou.

**Table:**

Country	Hospital name	Principal investigator
Austria	Christian Doppler Klinik	Eugen Trinka
	Landeskrankenhaus Salzburg	
	KH‐Hallein	
	Landeskrankenhaus Tamsweg	
	UKH	
Belgium	CHU Brugmann	Helene Visee
	Erasme Hospital	Chantal Depondt
	Cliniques Universitaires Saint Luc	Susana Ferrao Santos
	AZ Sint‐Jin Brugge	Annelies Van Dycke
	UZ Leuven	Wim Van Paesschen
Ireland	St James Hospital	Aleksei Gunko
	Sligo University Hospital	Cara Conway/Geraldine O’Rourke
	St Vincent's University Hospital	Cora Flynn
	Tallaght	Allan McCarthy/Denise Cunningham
	Beaumont	Abel Wakai/Masha Petricevic
	Cork	Daniel Costello
Sweden	Sahlgrenska	Johan Zelano
	Vasteras	Firal Al‐Jasami
	Karolinska	Sara Melin
	Norrlands	Mans Berglund
	Ostersund	Lisa Bergstrom
	Uppsala	Victor Vall
Netherlands	Maastricht University Medical Centre+	Rob Rouhl
Malta	Mater Dei Hospital	Nicola Dingli
France	Hospices Civils de Lyon	Sylvain Rheims/Karim Tazarourte
	CHU Nancy	Louise Tyvaert
	CHRU Lille	Phillipe Derambure/Marie Girot
	Bordeaux	Cecile Marchal
Norway	Haukeland Hospital	Eivind Kolstad
	Akershus Hospital	Silje Holt Jahr/Siri Hylleraas Bo
	St Olav's Hospital	Christian Samsonsen
Finland	Kuopio University Hospital	Reetta Kalvianen
Poland	Gdansk University Hospital	Maria Mazurkiewicz‐Beldzinska
Russia	City State Hospital by VM Buyanov	Igor Kaymovskiy
Switzerland	Centre Hospitalier Universitaire Vaudois (CHUV)	Phillipe Ryvlin/Ilona Wisiewski/Celia Bader
Germany	University Hospital Greifswald	Felix von Podewils
	University Hospital Marburg	Susanne Knake/Katja Menzler
	University Hospital Freiburg	Martin Hirsch/Jochen Brich
	University Hospital Aachen	Rainer Surges/Karmele Olaciregui Dague
	University Hospital Knappschaftskrankenhaus Bochum	Tim Wehner
	University Hospital Frankfurt	Adam Strzelczyk/Lara Kay
Croatia	University Hospital Zagreb	Lea Miklic
Greece	AHEPA University Hospital	Vasilios Kimiskidis/Martha Spilioti/Theodora Afrantou
	University General Hospital of Alexandroupoli	Aikaterina Terzoudi
	General Hospital ‘G. Papanikolaou’	Dimitrios Kazis
	University Hospital Ioannina	Sofia Markoula
	University Hospital Larissa	Katerina Markou
Cyprus	Nicosia General Hospital	Savvas Papacostas/Yiolanda Panayiota Christou
